# The Concept of Social Health From an Iranian Perspective: A Qualitative Exploration

**DOI:** 10.3389/fpubh.2022.797777

**Published:** 2022-05-10

**Authors:** Goli Soofizad, Sakineh Rakhshanderou, Ali Ramezankhani, Mohtasham Ghaffari

**Affiliations:** Department of Public Health, School of Public Health and Safety, Shahid Beheshti University of Medical Sciences, Tehran, Iran

**Keywords:** social health, qualitative study, Iran, adults, perspective

## Abstract

**Objective:**

As one of the health aspects, social health is less well-known than physical and mental aspects. In order to better understand this aspect and considering the importance of social context in its conceptualizing, the present study was performed aiming at explaining the social health and identification of its various aspects in the perspective of Iranian adults.

**Methodology:**

The present study was conducted in 2021 with a qualitative approach and with the participation of Iranian adults and social health professionals. Data were collected through semi-structured interviews with 36 participants who were selected by purposive sampling. The obtained data were analyzed using qualitative (conventional) content analysis and Granheim and Lundman method in the MAXQDA-2020. Guba and Lincoln criteria were observed to evaluate the quality of research results.

**Results:**

Using data analysis, 3 main categories and 17 subcategories were obtained, including: (1) Conceptual scope of social health (social health as social capital, social health as mental health, social health as moral health), (2) Characteristics of social health (biologic, continual, acquired, evolutionary, relative), and (3) Social health dimensions (openness to interactions, social adaptability, social dutifulness, social self-esteem, mutual trust, communicational capability, social optimism, enjoying social support, public-oriented personality).

**Conclusion:**

Since social health has a conceptual scope, it is important to try to strengthen and reproduce the dimensions of social health and at the same time use planning, policymaking and appropriate interventions to improve and to promote the dimensions of social health.

## Introduction

The concept of health is changing during the time. In the late 40s of the past century, the World Health Organization (WHO) defined health as “*a state of complete physical, mental and social well-being and not merely the absence of disease and infirmity”* (1). During the past decades, the concept of health has changed to become more dynamic and includes the ability to adapt and to self-manage in daily life (2). Social health is a concept that emerged in recent decades and is one of the dimensions of human health that plays an important role in his overall health and balance of social life, so that the results of several studies show that people with more social relationships have a 50% higher chance of survival than others (3). Lower social connections incur risks that are comparable to smoking and go beyond factors such as obesity and alcohol abuse, leading to premature mortality (4–7). According to various studies, social disconnection is associated with a 29% increase in coronary heart disease (8) and a 32% increase in the risk of stroke (9), dementia and memory loss in the elderly (10).

Several studies also show that during the COVID-19 pandemic and all previous pandemics, measures such as quarantine and social isolation, which prevented individuals from participating in society and social activities and having close contact with others, have had negative effects on people's mental health, including anxiety, and depression. Although social distancing policies have historically helped protect physical health worldwide, these policies have also greatly limited people's range of social interactions (11). Therefore, considering the importance of this dimension of health and its effect on the other two aspects of physical and mental health, social health is one of the priorities in the field of public health.

According to Vernooij-Dassen and Jeon (12), the added value of the concept of social health lies in several core features: it is an umbrella for an array of concepts reflecting human capacities to participate in social life, such as reciprocity and dignity, and resilience; it is a clear overarching concept facilitating communication between the psychological, social and biomedical sciences; it does not focus on deficits but on remaining capacities, and more importantly, it relates to normalcy (13, 14).

The concept of social health is far less well-known than physical health or mental health, while it is one of the three pillars of most definitions of health. Conceptualizations, albeit limited, have been made in the field of social health, and experts such as Block and Berslow, Donald, Huber and Keys have addressed the concept of social health and defined its dimensions (2, 15–18). However, the literature on social health is very limited and sometimes confusing, because this concept may be reliant on the situation and perception of individuals in any society, and it cannot be considered comprehensive, unified and universal. In other words, provision of a consistent definition of social health is difficult. Social health is a dynamic, fluid and context-based concept. This necessitates that the mental perceptions of individuals in society are considered to explain this concept and identify its dimensions, but in practice, this has not been the case. Although it's been more than seventy years since the WHO (19) first introduced three dimensions of human health, not enough attention has been paid to social health as one of the dimensions of health, and on the other hand, the context-based social health in a society like Iran with ethnic and cultural diversity intensifies the need to explain the concept of social health based on mental and experimental perceptions of people in society. With its relatively large population size and ethnic-cultural diversity, which is distributed in a relatively uneven and varied geography, has faced the health challenges in all of its dimensions. On the other hand, the society conditions, which indicate a transition from a relatively traditional to a relatively modern situation, bring opportunities and threats, i.e., opportunities to find solutions and models for the promotion and expansion of social health, as well as the threats of loss of existing social health or its decline with the cost of transition to a modern era (20). Due to lack of enough documents and approaches different social health assessments in Iran (perceived social health and objective social health), it is very difficult to make judgment about status of social health in Iran (21–23). Social health is one of the concepts that is difficult to define precisely and there is no formal definition of it in relevant literature (23). There are various definitions for it in this field which has led to different perspectives. It is not possible to accurately identify their correctness. On the other hand, social health is a concept that emerges in the context of society and interpersonal relationships in social networks. Therefore, in order to achieve a better understanding of the conceptual framework of this crucial dimension of health, according to the lived experience of citizens, qualitative research approach is a priority. The present study would be conducted for explaining of the social health concept and identifying its dimensions from the perspective of people and professionals so that by achieving a comprehensive conceptual framework and is innovative from this view. Accordingly, there is a fundamental concern and question as to; *How the concept of social health is defined from the perspective of individuals? What are people's perceptions and experiences of social health? What are the dimensions and components of social health?* A qualitative study can help to deeply explore and identify the hidden layers of people's lived experiences, interpretations and perceptions from the concept of social health.

## Methodology

### Design

This research used a qualitative approach and the conventional content analysis method. Participants included adults and social health professionals. Inclusion criteria were as being Iranian citizenship, willingness to participate in the study, adult age group (18–65) and physical or mental ability to participate in interview (lack of disabilities that interfere with communication, e.g., intellectual disability) for the adult subgroup as well as having expertise in the field of social health for experts. Subjects who did not have the above criteria were excluded from the study.

### Participants

In this study, purposive, and theoretical sampling with maximum diversity was used to select the participants. Sampling was performed with maximum diversity and according to the research objectives and questions to reveal the range of variations and differences in the field under study. On this basis, participants were selected from among men and women, different ages, different levels of education and different occupations, as well as from various fields of study including sociology, psychiatry, health education and promotion, epidemiology and community/social medicine. This part of study was performed in Tehran (for interviews with experts from all over the country) and all areas and neighborhoods of Urmia city, West Azerbaijan (for interviews with adults). Urmia is a multi-ethnic city with different religions and sects. Due to the ethnic diversity in Urmia, there is a lot of linguistic and religious variety and people with Turkish, Kurdish (Kurmanji, Sorani), Assyrian and Armenian languages as well as Shiite, Sunni (Hanafi, Shafi'i) and Christian religions have come together in this historic city (24, 25). Such a social and cultural environment provides a very good sample for studying social health on the adult population, which is a very context-based topic.

### Data Collection

To conduct the qualitative part of the study, after receiving the code of ethics (IR.SBMU.RETECH. REC.1398.081) from Shahid Beheshti University of Medical Sciences, the data were collected using face-to-face interview guiding questions and in-depth interview techniques over a period of about 8 months (from July 23, 2019 to August 21, 2020) by purposive sampling and the criteria for maximum diversity. Interview questions were slightly different for experts and non-experts (18–65 year old adults). Most of the interviews were conducted in Persian. Considering the fact that there were some Kurdish and Turkish participants, in cases where they did not want to be interviewed in Persian, a native language interviewer was used. The interview texts were analyzed after translation into Persian. The interviews were started with the following general questions; “*Since we, as humans, are social beings and interact with others in the society, we have a mix of trade-offs; we provide services to them and receive services from them. Since you are there in this community, how can your presence in the society become healthier?”* With this main question and the exploratory and supplementary questions of Table 1, the concepts, ideas, attitudes and meanings experienced by subjects and experts in relation to the social health concept were obtained.

**Table 1:** Checklist for questions of the interview guide.

**No**	**Questions**
1	How much do you know the concept of social health?
2	How do you think a person's social health is defined?
3	How a person could become healthier in interaction with society and individuals?
4	Who do you consider to be socially healthy/unhealthy?

The participants were then guided and interviews continued based on the contents and experiences. The interviews were immediately transcribed and analyzed. By simultaneously analyzing the data and obtaining the concepts and categories, more questions were provided for the researcher to be used in subsequent interviews, that is, the form, order, and subject matter of the questions changed at different stages of the study as the data collection process continued. Interviews were conducted in public spaces, parks, workplaces and where the participants agreed with. The average interviews lasted 45 min (maximum 70 min and minimum 20 min) and were conducted in a quiet environment having sufficient light, no noise and without the presence of other people. In total, 36 interviews were conducted, of which 29 were conducted with adults using purposive sampling and 7 with experts *(3 PhD in Sociology, 1 PhD in Psychiatry, 1 Clinical Psychology Doctorate, 1 PhD in Public Health, 1 in Area of Epidemiology, 1 PhD in health education and Promotion)*. The demographic characteristics of the adult interviewees are listed in Table 2. Data collection continued until saturation. That is, when interviews no longer added anything new to the findings and the content and codes became duplicates. The codes became duplicates in 29 interviews; however, the researchers continued the interviews up to 36 subjects for more certainty and to prevent false saturation.

**Table 2:** Demographic characteristics of adults' participants in the study.

**Variable**	**Groups**	***N* (%)**
Gender	Female Male	14 (48) 15 (52)
Age group	<30 30–50 > 50	6 (21) 15 (52) 8 (27)
Marriage status	Single Married Divorced	12 (41) 15 (52) 2 (7)
Education	No formal education. High School and Lower Diploma Higher Education	1 (3) 4 (14) 6 (21) 18 (62)
Employment status	Housewife Unemployed Employee Self-employed	4 (14) 3 (10) 12 (41) 10 (35)
Ethnicity	Persian (Parth) Kurd Turkish Assyrian	4 (14) 9 (31) 11 (38) 5 (17)
Religion	Shiite Sunni Christian	13 (45) 11 (38) 5 (17)

### Data Analysis

Data analysis was performed using the 5-step method of Granheim and Lundman (26) and using MAXQDA.V.2020 software. In the first stage, the researcher transcribed the interviews on the same day after conducting the interview. In the second stage, the transcription of the interviews was reviewed several times to obtain a general understanding. The transcription of the interviews was reviewed word by word to obtain the initial codes. In the fourth step, the researchers classified codes that were similar in terms of meaning and concept in the same category, and also determined how they are related. Then, in the last stage, the categories formed in the previous stage were placed in more comprehensive and abstract classes, and the themes were obtained. Two members of the research team collaborated in data analysis phase (including the first author and the corresponding author of the article). After the final analysis, all steps were explained in one session for all authors of the article, and in some cases, the categories and subcategories were renamed.

### Trustworthiness

To increase the credibility and quality of the results, the four criteria of Guba and Lincoln were observed (27–29). To gain credibility, the findings were provided to the participants and the categories and subcategories were approved. The researchers also selected participants with the greatest differences in terms of demographic characteristic. In order to obtain confirmability, the coding, categorization and reporting methods of the results were approved by 2 experts in qualitative research. To increase dependability, all authors monitored the research process carefully and expressed their views on the findings and how they were coded during online sessions. In order to enhance the transferability of the study, a complete description of all stages of the study was provided and a large number of direct quotes were expressed from the participants.

### Ethical Considerations

In order to observe the ethical codes and principles of the study, all participants provided informed and written consent, and were assured that their information would be disseminated only in line with the objectives of the study and that data such as recorded interviews would be deleted after the study was completed and the participants' quotes will not be evaluated and judged. They were also told that participation in the study was completely voluntary and that they could leave the interview at any time.

## Findings

This study was performed by interviews with 36 subjects and, after analysis, the results were classified into three main categories and 17 subcategories.

### Conceptual Scope of Social Health

Because social health is a complex and not so well-known concept compared to physical and mental health, in interviews with individuals, social health was found to have a range of concepts that are sometimes synonymous with social capital or the social elements of mental and moral health (see Table 3).

**Table 3:** Categories, sub-categories and codes obtained from interviews with participants.

**Description of coding category**	**Sub-categories**	**Category**
Social health is a double-sided tape that maintains the individual and society together, social health causes and creates social capital, social capital that produces social health, mutual relationship and trade between the two concepts of social capital and social health, social capital is the same social health	Social health as S*ocial Capital*	Conceptual scope of social health
Mental health is the underlying factor of social health, perfect mental health is the same as social health, considering both individual and social elements for mental health, social health at the same level of mental health in the social field, social health follows up positive mental health movement, social health is related to both the individual and the society	Social health as *Mental Health*		
Moral health is the same as social health, alignment of social health and moral health	Social health as *Moral Health*		

#### Social Health as Social Capital

Social capital is a concept that is very close to social health and this common conceptual space is expressed in different ways by participants, i.e., social health that produces social capital, social capital that produces social health, as well as trade-offs and mutual relations between these two concepts, which can be clearly seen in the views of participants.

“*In his 5D model, Keys talks about social cohesion, social participation, social acceptance, social adaptation, and ultimately social actualization. These are undoubtedly components of social capital, such that institutional participation and non- institutional participation, individual trust and institutional trust as well as social cohesion and coherence, and social support, as the five components of social health overlap with the dimensions of social health as expressed by Keys” (Male, Sociologist)*.

Social health and social capital overlap in many of their components, and there is a mutual relationship between these two concepts. Therefore, social capital increases and strengthens social health, and social health also follows the emergence of social capital. But more importantly, many components of social capital and social health, including participation, coherence, trust and support are common between the two.

#### Social Health as Mental Health

Many participants considered social health in their mental health assemblage. Mental health is not just the lack of disorder but also a range of individual and social characteristics such as the ability to cope with daily stress, being useful and productive in assigned tasks, and the ability to participate in the society.

“*The social health of an individual is actually the mental health is in his social sphere. There is a time when I am aggressive and aggression occurs only in relation to others. This is social health. If I am a pessimist and say society is not trustworthy, this is the social aspect of mental health. Pessimism, although related to mental health, is concerned with our social relationships. I'm shy when I'm a recluse. That is, I avoid being in society. This is related to the social part of mental health. In other words, when mental health is related to our social behavior, it becomes the social health at the individual level” (Male, Psychiatrist)*.

Accordingly, perfect or positive mental health includes the components of individual social health and the social health is one of the two main dimensions of mental health and oversees the type of behavior and reaction of the individual in interaction with others.

#### Social Health as Moral Health

Moral health is a concept that some participants equate to social health. From their point of view, social health is the do's and don'ts that every individual, as a social being in society and in interaction with others, should consider and adhere to.

“*Morality and social health are integrated and cannot be separated. As said, what the Shari'ah dictates, so does the intellect. In social health, we have the obligation not to insult others and to respect them as they are. We should not blame others due to their dressing. It means that we should accept everyone as he is, i.e. to listen to him morally and look well and be receptive and respective and help him if we can” (Male, 47, University Professor)*.

Social health and moral health are in the same direction and intensify each other, so that the weakening of morality affects the way people connect to each other in society and ultimately have an increasing and reciprocal effect on each other. Observing minimum ethical principles, such as justice and collective interests and avoiding selfishness, and generally respecting society and the moral principles defined in the society, all testify to the intertwining of the two concepts of social health and moral health.

### Social Health Characteristics

Another important finding of this study includes the characteristics that the participants mentioned for the concept of social health that defines a specific framework for this concept, including that social health is a biological, evolutionary, continuous, acquired and relative concept (see Table 4).

**Table 4:** Categories, sub-categories and codes obtained from interviews with participants.

**Description of coding category**	**Sub-categories**	**Category**
Socialization is a necessary trait for survival, biological nature of social health, social health is a part of individual health	Biologic	Characteristics of social health
Being in the middle of the continuum of individualism and collectivism, balance in individual and social life, not confining social health only to relationships between individuals	Continual		
Correct education, learning how to treat others throughout life	Acquired		
Development of altruism of humans toward animals and the environment over time, evolution of attachment, evolution of trust, evolution of the sense of responsibility	Evolutional	
Lack of a universal index for social health, dependence of social health on the social context, dependence of the definition of social health on the disciplines of experts, dependence of social behavior on time and place	Relative	

#### Biologic

According to some participants, social health is a biologic feature in humans and socialization, as one of the most important components of social health, is a necessary trait for survival. This biologic feature is more transparent in some groups, including Iranians.

“*Social health has a biologic basis, that is, it is not a concept made up of nothing, I think a part of the brain is related to this type of attachment. It's been created for survival, people survived because of attachment. I mean, the nature has chosen those who have had the instinct of attachment, I believe a large part of our socialization process in Iran has been biological. I think it is much less in Europeans. That means we would love to belong to a group” (Male, Social Medicine Specialist)*.

“*Social health is a part of a person's health, so, just as we measure physical health with a thermometer, we measure a person's mental health with some criteria. So, using some criteria we can measure one's social health” (Male, Epidemiologist)*.

Social health was recognized as a concept with a biologic basis and is not essentially a constructed and abstract concept. As one of the dimensions of health, and like other physical and mental dimensions, social health has objective components and can be measured by related criteria.

#### Continual

Social health is not a definite point, but a continuum. Everyone, based on his/her proximity to the two ends of the spectrum, i.e., the degree of individualism or collectivism, benefits from social health differently.

“*Being social is a continuum that can be harmful on both ends of the continuum. If you are very social, it will harm you. You have to conform to your social needs. If you are too far away from the community, you will harm others. You hurt on the one end and get hurt on the other. In this social continuum, the middle ground must be chosen” (Male, 43 Years Old, Teacher)*.

“*The point of moderation in the continuum of health is that I gain and grow and behave in a way that does not harm myself or others” (Female, 44, University Professor)*.

In the social health continuum, when someone is in the middle point of the continuum, it means that the person has a balanced social life.

#### Relative

Social health, due to its social nature, is very dependent on time and place and has special components in every cultural and social context.

“*A citizen who participates, trusts and is trustable is a citizen who has social health, a citizen who adapts to and cooperates with the society. This is the link between society and the individual in terms of social health, which can vary in different communities. For example, a society defines attachment in such a way that marriage takes place and farmers and gardeners help each other. In another society, the type of attachment and behavior may be different. Therefore, you can consider a level of specific norms of that society as the realization of social health, which does not contradict the generally accepted norms of society. The same is true of the passage of time. Concepts that have emerged in Iran in recent years, such as a sense of social belonging, could not be seen before” (Male, Sociologist)*.

Social health lacks a general universal index and is highly subject to both physical and mental dimensions. On the other hand, due to the lack of a comprehensive definition, in each specialized field, social health is defined in a different way and experts in each field have relied on their specialized disciplines in defining this concept.

#### Acquired

Although human beings are inherently social beings, they acquire social health through social interactions with family, friends, and the community over time.

“*Social health is a relative concept, the result of a proper education. A person who is not socially healthy from the beginning could learn it from family, friends, and society. I mean a person gains social health over time” (Male, 32, Driver)*.

“*The issue of socialization, although intrinsic in the individuals, may grow by the environment and surrounding individuals” (Female, 39, Teacher)*.

Education is one of the methods to achieve social health. During socialization in the context of family, friends and society, one learns and internalizes many components of social health.

#### Evolutional

Social health is a completely evolutionary concept, the dimensions of which have gradually expanded over time. So, altruism, the desire for social relations and a sense of responsibility, have been primarily related to humans and have gradually spread to animals and the environment.

“*In my opinion, altruism and social relations have been primarily related to human beings, and it is slowly expanding to the environment and animal protection, so that they could also help us. And this is projected on different things and is given a social meaning. For example, the sense of responsibility has been an evolutionary trait. The society did something for the person, and the person thought he/she should have done something for the society. Communities with this type of people have survived more and this has become a social value” (Male, Social Medicine Specialist)*.

“*If you look closely, you can see that in the past, people had a limited circle of communication, only being in contact with their own family, tribe and clan, and trusting only these people; but today, with the increase of human knowledge, fear of strangers has decreased and people sometimes trust someone on the other side of the world and do not worry. Thanks to technology and the virtual world, I have made new friends on the other side of the world and we share our experiences with complete confidence” (Female, 74, Retired)*.

Social health has evolved over time and its components, including trust and the sense of responsibility, have expanded. As humans have evolved over time, societies have found that they may survive longer if they are able to do something for others and work together, and thus the sense of responsibility and cooperation has developed over time. On the other hand, increased knowledge along with increased sense of altruism among human beings expanded trust beyond the limited circle of acquaintances.

### Dimensions of Social Health

Participants defined multiple dimensions of social health that based on the specific characteristics and skills of the individuals in relation to others and society, based on which individuals' social health can be assessed, these dimensions are: *Openness to Interactions, Social Adaptability Social Dutifulness Social Self-esteem, Mutual Trust, Communicational Capability, Social Optimism, Enjoying Social Support, People-oriented Personality* (see Table 5).

**Table 5:** Categories, sub-categories and codes obtained from interviews with participants.

**Description of coding category**	**Sub-categories**	**Category**
Being sociable, sense of social belonging, ability to maintain existing social relationships, not retaliating the wrong behaviors, maintaining family kinship, having good relationships with relatives and friends, being able to make new connections, being able to have quality relations	Openness to interaction	Dimensions of social health
Acceptance and tolerance toward others, empathy with others, normal socialization	Social adaptability		
Responsibility in all social roles, active participation in society, respect for the rights of others, prioritizing the common good	Social dutifulness		
Self-confidence in expressing views, protesting against bad behaviors, recognizing one's position	Social Self-esteem	
Honesty in relation to others, trustworthiness, trust in others, trust in social institutions	Mutual trust	
Being open to criticism, speaking and acting appropriately	Communicational capability	
Positive thinking about society, life satisfaction	Social optimism	
Gaining the support of friends and family under special conditions, ability to benefit from the support of social institutions	Enjoying social support	
Kindness to others, resilience in stressful situations, peace of mind in relation with others, being accepted by others, respect for others	People-oriented personality	

#### Openness to Interactions

One of the components of social health is the state of openness to interactions, such factors as being social, being more in touch with the society, having a sense of social belonging and loving society and people, in addition to maintaining existing social relationships, ability to enter new relationships and have effective and quality communication with others.

“*The fact that people love their neighborhood, community or people is a sign of a sense of social belonging and this, in a way, goes back to their social health” (Male, Sociologist)*.

“*Creating friendly relationships with others is very important, we have to maintain relationships with others we know as well as make new friends” (Female, 39, Teacher)*.

“*It's not just about connecting with others. We need to be able to have a good time with others and enjoy being in a group” (Male, 47, University Professor)*.

There are different degrees of sociality in different people, which leads to relationships with others. Also, the sense of belonging leads people to have more contact with society. Human beings are in contact with many people due to their membership in different familial, kinship, educational, and occupational groups. The ability of individuals to maintain these existing relationships is itself a capability that reflects the social health of individuals. One of the behaviors related to maintaining existing relationships, especially in Iranian-Islamic culture, is maintaining family kinship. A socially healthy person has abilities based on which he can have acceptable relationships with people of the same or other generations or people belonging to the opposite sex. In addition to maintaining existing relationships, individuals must have the ability to make new connections and expand their network of friends. Finally, a socially healthy person, in addition to maintaining existing relationships and making new friends, has quality connections, so that he/she has satisfactory connection with others, enjoys being in a group and tries to have fun moments with others.

#### Social Adaptability

Another component of social health is social adaptability, which refers to some traits in people, such as being tolerant and receptive toward others, having the ability to accept others with any beliefs and tastes, having an empathetic spirit and understanding others far from any judgment. Finally, individuals should have the power to adapt to the codified and non-codified laws of their society.

“*A socially healthy person is one who accepts others in any situation based on mutual respect and away from any bias and prejudice. People enter society with different tastes and mindsets, people must be able to adapt” (Female, 41, Employee)*.

“*We have to respect our norms completely and should not break the law. The better people understand and follow the rules and regulations, the more they would show signs of social maturity and they are more socially healthy” (Female, 26, Housewife)*.

Therefore, away from any bias and prejudice, a socially healthy person, respects the opinions of others and understands others without any judgment. Also, because norms and abnormities are different in each society, a socially healthy person would have a clear understanding of the norms of his community. Healthy behavior in the society is a behavior that is in line with these values and norms. Therefore, the more an individual can understand the codified and non-codified laws of the society and update his understanding, the more socially healthy the person will be.

#### Social Dutifulness

Social dutifulness has been recognized as another dimension of social health, which refers to the observance of the rights of others and respect of justice, the priority of collective interests, responsibility toward society, and participation in the society.

“*Social health, in my opinion, requires respect for the rights of others, that is, treat others as you would like others to treat you” (Male, 54, School Principal)*.

“*Disregarding others, sticking to oneself and one's own life, one's baby, one's mother, this means self-centeredness. Self-centeredness is at odds with social health because, in my opinion, social health means the wellbeing of oneself and others” (Male, Psychiatrist,)*.

“*In any role, if you are a good actor, you are a healthy person. It means everyone should know the role he/she has accepted, does not slack off and meets the needs of people” (Female, 39, Teacher)*.

“*An important criterion for a socially healthy person is how much he/she participates in social affairs and the public good” (Male, Epidemiologist)*.

A person who cares about the common good, respects the rights of others, has a sense of responsible for his duties as well as toward those around him and his community, and participates in social activities, is a socially dutiful person, and therefore he can also enjoy social health.

#### Social Self-Esteem

This aspect of social health refers to traits such as self-esteem in expressing views, recognizing one's status and protesting against the bad behaviors of others. These are traits that protect an individual in his/her social interactions.

“*The point of moderation in the continuum of health means I have to grow and behave in a way that does not harm myself or others. I should eliminate neither myself nor others” (Female, 44, University Professor)*.

“*People should realize that they have a right that others should respect. It means that you, as a citizen, have a right, and in return you have a duty that you have to perform. If we do not observe these, we cannot live along with each other. Therefore, a person must know and be aware of his rights” (Male, 36, Employee)*.

Therefore, in interaction with others and in addition to respecting the rights of others, members of society should also value their own rights and privacy and be aware that in these interactions and trade-offs with society, they should not exclude themselves; they should be able to easily express their views; protest against the wrong behaviors and words of others, and defend their rights in the right way. Socially healthy people have peace of mind and Self-esteem in doing things as well as in dealing with others. In this way, communication leads to healthier and more lasting interactions, because it leads to a greater sense of satisfaction and relaxation in the relationship.

#### Mutual Trust

Mutual trust is another dimension of social health that includes honesty and integrity, avoidance of sneakiness, trust in others, being trustable and having a positive attitude toward others and trust in social institutions.

“*In my opinion, in order for someone to be socially healthy, he/she must first be honest and right-doing, otherwise he/she will not be able to communicate easily with others and no one will take his words into account” (Male, 32, Driver)*.

“*A citizen who trusts and is trusted is a citizen who is socially healthy” (Male, Sociologist). “I clearly see that families no longer trust school and society in establishing justice for their children, and in raising their children, they raise them in a selfish way” (Male, 54, School Principal)*.

One of the most important components of trustworthiness is honesty and integrity. Secretiveness (Sneakiness) and disregarding honesty damage social interactions. A person's behaviors are easily accepted by others due to his honesty, right doing and predictability, and, he can have lasting and strong relationships with others. On the other hand, trust in others as well as in social institutions is one of the most important characteristics that leads a person to cooperation and social participation and makes a person consider himself belonging to, and part of the society.

#### Communicational Capability

Another dimension of social health is communicational capability. This dimension of social health indicates the extent to which a person is proficient in speaking concisely and at the same time comfortably and powerfully, is open to criticism and can think about it, and he/she can manage his/her behavior in a way that is commensurate to a social context and situation.

“*Socially healthy people have a high level of oratorical skills. He/She expresses his/her beliefs very concisely. The more eloquent a person is, the more attractive he/she will be. Communicating with others is an art in itself. A person who communicates easily with others has communication skills and will be successful in his/her relations” (Female, 22, Student)*.

“*One can have a state of receptiveness, reception and learning. One who is a good critic must also be open to criticism. One could not be a critic and do not accept criticism” (Male, 39, Teacher)*.

“*It means if any of my behaviors and dialogues and any of my actions and needs can be helpful or harmful to me according to the cultural and civilization needs of society. This is a trade-off between the behaviors so that the person can make decisions in the face of social situations that neither harms himself nor others” (Male, 43, Teacher)*.

Because dialogue is the beginning of any communication, it is very important how the person expresses his words, in what tone, long or short. On the other hand, despite the desire for approval, individuals and their actions are not always approved. This is a basic communication skill that people can be open to criticism and are ready to think about it. It is also necessary for a person to manage his behavior in society and in his social interactions in a way that is appropriate to that context and situation of the society, so as not to harm society and the people who are in contact with him.

#### Social Optimism

Another dimension of social health is social optimism, which refers to an attitude in the individual that is based on positive thinking, focusing on the good, opportunities and possibilities instead of negative points and threats in society, as well as life satisfaction and hope for the future.

“*I believe that the more positive I think, the better my life and my relationship with others” (Female, 58, Gym Coach)*.

“*In my opinion, social health is the type of attitude and perspective of an individual toward society. Socially healthy individuals have hope for the future, do not give up too soon, always try and are motivated” (Female, 41, Employee)*.

“*People who think they are useful for the society will be satisfied with themselves. These people are acceptable to others and are always satisfied with the results of their behavior and have hope for the future” (Male, 26, Self-employed)*.

The type of attitude toward the community and friends will be very effective in the individual's readiness to interact and get in touch with the community. Positive and happy individuals not only enjoy their interactions and relationships, but also are satisfied with life, and this life satisfaction affects their performance which presumes this cycle of satisfaction and good performance.

#### Enjoying Social Support

Another dimension of social health is enjoying social support, which means having the support of family and friends in certain situations such as illness, emotional or financial problems, and the ability to benefit from social institutions.

“*It is very important to have a trustable mate in a difficult situation, to speak your heart out, and may get help if you get sick” (Female, 53, Gym Coach)*.

“*I think one should be able to count on others for help and support in certain situations such as illness or financial problems. It is very difficult to think that you can solve all the problems alone. In this situation, I am not ashamed to ask for help from my family and close friends, because I will also help them if this happens to them” (Male, 56, Retired Judge)*.

“*In my opinion, social health is the use of the components of a healthy society or a balanced and cohesive society. One would benefit from and use these components taken from the society to promote his personality, psyche and individuality” (Male, Sociologist)*.

People may face various crises in life, including illness, emotional or financial problems. In such a situation, people who can benefit from their emotional support are better able to cope with the challenges ahead. On the other hand, these people should have a spirit in such a way that they refer to friends and relatives and try to attract their support and help. Communities support individuals at different levels broadly or to limited extent in the form of social institutions. Individuals should have the ability to use data, opportunities, and community support to enhance their personality, psyche, and individuality.

#### People-Oriented Personality

The last dimension of social health is people-oriented personality, which implies personality traits in individuals that indicate social health. These traits include the power to influence others, resilience, composure and calmness, kindness and respect for others.

“*Selfish individuals cannot influence others, and it is very important to know to what extent one can influence others. In this market, I know people in whom I trust very much and are very trustworthy in every way. I think they are of high social health” (Male, 41, Self-employed)*.

“*Socially healthy people are calmer and more relaxed. Socially unhealthy ones, on the other hand, are angrier and aggressive. These people fly off the handle with the slightest stimulus” (Female, 43, Housewife)*.

“*Kindness is the basis of social health. That means a person should be kind, seeks the good and health of others, does not envy others, prays for all and treat people with kindness” (Female, 38, Teacher)*.

“*When we are socially healthy, we should not mock someone who says something that we don't like. Communication between individuals should be based on mutual respect” (Female, 18, Student)*.

Social health requires a personality whose attitudes and behaviors are people-oriented. A people-oriented person has the power to influence others, is a respectful person, and is patient and calm. He/She is far from stress and aggression and has high resilience. He/She is kind and benevolent and spreads kindness.

## Discussion

The present study aimed at explaining the concept of social health and identifying its dimensions from the perspective of Iranian adults. A summary of the results of each category and subcategories is presented and a discussion is provided on the conceptual scope of social health, characteristics and dimensions of social health. It was found in this study that social health has a wide range of concepts and due to the lack of a clear theoretical framework, it sometimes equates with social capital, moral health or mental health. Consistent with our study, Amini Rarani et al. (30) and Sharbatian (31) explained that about 30 and 59% of the social health variance is determined by the social capital, which somehow indicates the similarity of the conceptual space between these two variables. On the other hand, indicators such as social contribution, social actualization and social coherence and social acceptance as dimensions of social health in Keys theory can be considered the psychological capital (32), meaning that the dimensions of social health introduced by Keys are in fact the cognitive social capital. On the other hand, the results of this study show that mental health and social health strongly overlap despite the fact that they are defined as two separate dimensions of health. In line with our findings, in most definitions of mental health, including the definition of the WHO (33), how a person is present in the society is part of mental health. Keys also considers social well-being as one of the three components of mental health (17, 34). Moral health is another concept that is sometimes considered equivalent to social health in this study. Accordingly, social health is the do's and don'ts that every individual should adhere to as a social being and in interaction with others. In line with this finding, Durkheim believed that moral phenomena are, in a way, subsets of social phenomena, and morality of the human being is the result of his socialization. According to Durkheim, ethics means that an individual is related to others in the society and social cooperation is established between them (35, 36).

Another finding of this study is the five features of social health. Social health is a purely biologic attribute, part of individual health, and is rooted in human instinctual socialization. Social health is more a biologic and measurable concept than an abstract one. Social health is a relative and contextual concept and is defined in various ways at different times and places. Social health is a continuum, i.e., it cannot be said that a person is completely socially healthy or lacks social health; we can only say where a person is in the continuum of social health. Social health is an acquired asset and one learns it over time and in the process of socialization. Social health is a concept that has evolved over time, so that responsibility and trust have expanded over time as its components from and family members and relatives to the larger community and the world. In the social health literature, no study was found to be consistent with or opposite to this finding, and it can be said that these characteristics are new findings obtained in our study.

Another important finding of this study is identifying the dimensions of social health. In this study, nine dimensions were identified for the social health. One is *Openness to Interactions*, which includes concepts such as sociality, sense of social belonging, ability to maintain existing social relationships, ability to establish new relationships, ability to have quality communication. Consistent with our study, numerous studies have identified similar components for social health, including; social interaction (37), social interaction and family relationship (38), relation with the relatives (39), social contact frequency and social networks of individuals (40), marital quality and relationship strain (40), relationship satisfaction (41), meaningful relationships (2), social integration and sense of belonging to society (32, 40). The abilities to maintain existing social relationships as well as to establish new relationships indicate the other two variables of openness to interactions. The importance of these two dimensions is that a person who cannot communicate with family, friends and society is socially disabled. Findings of Green et al. study confirms this result (42). *Social Adaptability* is another dimension of social health that was identified in this study. This dimension includes three concepts of reception and tolerance toward others, empathy and normative socialization. Consistent with our findings, one of the dimensions of social health in the study of Keys is the social acceptance (18). Also in Rafiei's study (38) empathy is known as one of the components of social health. Normative socialization is one of the new findings of this study, which means understanding the norms, values, and codified laws of society, being aware of their usefulness, and respecting and observing these norms and values. Another dimension of social health in this study is *Social Dutifulness*, which implies respect for the rights of others, prioritization of collective interests, responsibility, and participation. The results of similar studies also include variables such as conscience, social responsibility, social participation (38), participation in social groups and decision-making, environmental protection (39), and social contribution (18). In another study by Hahn et al. (41), participation in occupational, social, and family roles is a key component of social health. In conceptualizing positive health (2), one of the dimensions has been the social participation of the individual, which in addition to social presence, also includes active participation in society. *Social Self-esteem* was another dimension of social health in this study, which indicates self-confidence in expressing views, recognizing one's status, and protesting against the bad behaviors of others. As another dimension of social health, *Mutual Trust* includes honesty and integrity, trustworthiness, trust in others, and in social institutions. Consistent with the findings, confidence in the intrinsic goodness of others and a positive look toward humans and believing in the well-being of people are the hallmarks of social acceptance in Keys' study (17, 18, 32, 34, 43). *Communicational Capability*, as another dimension of social health, refers to the capacity of the individual to speak well, be receptive and act in accordance with the context. The results of Huber et al. (2) indicated that the communication and social skills are other variables of social participation. Another dimension of social health is *Social Optimism*, which indicates a kind of positive thinking toward society and life satisfaction. In the study of Rafiei et al. (38), the attitude toward society is one of the main dimensions of social health. The dimension of social actualization in Keys' theory also confirms this finding (44). As another dimension of social health, *Enjoying Social Support* is of two levels. The fact that at a micro level, the person can count on family and friends for help and support in certain situations, and, on the other hand, he/she has the spirit to attract the support of family and social institutions. In this regard, several studies emphasize the importance of having the support of others (family, friends, and relatives) and the society (39, 40). Social health requires a *People-oriented Personality*, which is another dimension of this concept and refers to characteristics in the individual such as the ability to influence others, resilience, composure, calmness, kindness, and respect for others. As one of the variables of social health, kindness includes financial and emotional support, kindheartedness to and smiling at others, which has been obtained in similar studies (38, 39). Respect for others, resilience, composure, and calmness are among the prominent personality traits in a socially healthy person which have not been mentioned in similar studies.

Reviewing the interventions in the field of social health in Iran, it was found that a small number of interventions has been performed in this field and in most of them, the promotion of social health has been a side goal (45–48).

Studies have been performed throughout the world that could be a good guidance to enhancing the social health components identified in this study. Specifically, regarding *Social Adaptability*, interventional studies such as book reading sessions aiming at enhancing the intra- and inter-generational relations (49), as well as community health workshops among peer groups aiming at improving adaptability potentials of immigrants in the new society (50) seem to be well-established interventions. Designing interventions for group-based physical exercise and increasing vitality at work (51, 52) could enhance the *openness to interactions* of individuals. Increasing civil partnership through methodologies such as socio-therapy by redesigning the values, norms, relations, and possible collaborations (53) are examples of interventions that seem to be applicable for the improvement of *Social Dutifulness*. Also, interventions aiming at the enhancement of social participation and social support, especially in the elderly (54), so that they could cope with the loneliness, are examples of interventions that are commensurate with the identified aspects in this study.

Therefore, it seems that interventions aimed at promoting effective communication, social capital, perceived social support, altruism, and social integration can help promote social health. Group discussions, socio-therapy, and physical group activities can also be effective ways to perform these interventions in neighborhoods or organized settings, such as workplaces, universities, and cultural clubs for adult.

## Limitations and Strengths

One of the strengths of this study is investigating the social health which has received less attention than physical and mental health. Also, based on enquiry and review of various resources and researches by the authors, it was found that the present study is one of the few studies on social health in Iran and the world that has investigate this concept in a qualitative way from the perspective of two subgroups of people and experts. Such a conceptualization of the social health based on the views of Iranian society could pave the way for a more accurate assessment of this dimension of health in society and provide useful data for policy makers and social health activists in order for planning, policy-making and appropriate interventions. But this study also had its limitations: the ambiguity and disagreement about the concept of social health and the lack of favorable cooperation of the participants in the face-to-face interviews, mostly due to the COVID-19 pandemic and traffic restrictions, created difficulties in obtaining appropriate information and data.

## Conclusion

According to the results of this study, social health is a completely relative, evolutionary and biologic concept that has an acquired and continuous character. While having a common conceptual space with areas such as social capital, moral health and mental health, the social health has special dimensions such as openness to interactions, social adaptability, mutual trust, social self-esteem, communicational capability, social dutifulness, social optimism, enjoying social support and people-oriented personality. Considering the obtained results and the importance of social health characteristics and its various dimensions, it is important to strengthen and reproduce the dimensions of social health and at the same time, plan, make policies and appropriate interventions to promote and strengthen the dimensions of social health. Achieving a better understanding of the conceptual framework of social health and its dimensions in the context of Iranian society, helps to design appropriate tools for measuring social health that can be used by researchers and policy makers in this field.

## Data Availability Statement

The raw data supporting the conclusions of this article will be made available by the authors, without undue reservation.

## Ethics Statement

The study was provided ethical approval by the Shahid Beheshti University of Medical Sciences (IR.SBMU.RETECH. REC.1398.081). In order to observe the ethical principles of the study, all participants provided informed and written consent.

## Author Contributions

GS and MG involved in data collection. GS, AR, SR, and MG reviewed and involved in data analysis. SR, GS, and MG reviewed the first draft of the manuscript. All authors approved the final version of the manuscript. All the researchers contributed to the concept and purpose of the study.

## Conflict of Interest

The authors declare that the research was conducted in the absence of any commercial or financial relationships that could be construed as a potential conflict of interest.

## Publisher's Note

All claims expressed in this article are solely those of the authors and do not necessarily represent those of their affiliated organizations, or those of the publisher, the editors and the reviewers. Any product that may be evaluated in this article, or claim that may be made by its manufacturer, is not guaranteed or endorsed by the publisher.
